# Effect of helicobacter pylori infection eradication on serum level of anti-tissue transglutaminase in children with celiac disease

**DOI:** 10.1186/s12887-023-03934-1

**Published:** 2023-03-08

**Authors:** Pejman Rohani, Maryam Monajam Zadeh, Hosein Alimadadi, Mohammad Hassan Sohouli

**Affiliations:** 1 Pediatric Gastroenterology and Hepatology Research Center, Pediatrics Centre of Excellence, Children’s Medical Center, Tehran University of Medical Sciences, Tehran, Iran; 2 Department of Pathology, School of Medicine Pediatric Chronic Kidney Disease Research Center, Childrens Medical Center, Tehran University of Medical Sciences, Tehran, Iran; 3 Student Research Committee, Department of Clinical Nutrition and Dietetics, Faculty of Nutrition and Food Technology, Shahid Beheshti University of Medical Sciences, Tehran, Iran

**Keywords:** Celiac, Anti-tissue transglutaminase, Helicobacter pylori, Children

## Abstract

**Background:**

Evidence shows the increase of anti-tissue transglutaminase (tTG) levels in various conditions, including infectious agents, independently of celiac disease (CD). The aim of this study was to investigate the effect of helicobacter pylori (H.pylori) infection eradication on serum level of tTG in children with CD.

**Methods:**

This study was conducted on children aged 2 to 18 who referred to reference hospitals for diagnosis of CD. After upper endoscopy and biopsy to confirm CD and H.pylori infection, the children were divided into three groups (including group one: 16 CD patients with positive H. pylori; group two: 16 non-CD patients with positive H. pylori; and group three: 56 CD patients with negative H. pylori), respectively. The tTG level in study groups were compared after the eradication of H.pylori.

**Results:**

The mean age of the subjects in the group one, two, and three was 9.7 ± 3.33, 11.8 ± 3.14, and 7.6 ± 3.32 years, respectively. Our results showed that in group one, mean tTG increased after eradication of H.pylori infection, however, these changes were not significant (182.43 vs. 157.18, P = 0.121). In the second group, although unlike the first group, mean tTG decreased after eradication of the infection, but still these changes were not significant (9.56 vs. 22.18, P = 0.449). Furthermore, at the baseline level, the mean tTG in the group three was closer to the mean tTG in the first group.

**Conclusion:**

Our findings showed that the eradication of H.pylori infection does not have a significant effect on tTG levels in children with and without CD.

## Introduction

Celiac disease (CD) is known as an immune-mediated disease that can develop in susceptible individuals after exposure to gluten [1]. CD is the most common enteropathy affects 1% of population worldwide [2]. CD is characterized by a set of serologic features, histological changes in intestinal tissue, and gluten-related intestinal and extraintestinal symptoms that can vary from person to person [3]. However, in order to screen this disease in the general population and population at high risk of IgA and symptomatic, it is recommended to use a very sensitive test called Anti-tissue transglutaminase (anti-tTG) IgA antibody, which like other immune-mediated disease, mechanism of anti-tTG production in patients with CD has been precisely determined [3, 4].

In recent years, some evidence shows the role of infectious agents in the development of autoimmune diseases, including CD, as well as some of the characteristics of this disease, including increased anti-tTG production [5, 6]. So that it has been suggested that infectious agents increase this antibody through the conversion of tTG and gluten peptides into macromolecular aggregates and as a result tissue changes related to gluten [6, 7]. In the study of Ferrara et al., it was also shown the independent role of these infectious agents in the production of anti-tTG antibodies [8]. It was also stated in this study that anti-tTG produced by infection have the same effect on tissue changes, inflammation, and other damages in the body [8].

H.pylori infection is common infection especially in developing country [9]. The precise prevalence of pediatric H.pylori and CD is not defined in Iran. But it seems that both conditions are prevalent [9]. A lot of study was performed about association of H.pylori infection and CD and different conclusion were achieved [10–12]. So that, it has been shown that H.pylori infection and CD have overlapping pathological features [10, 12, 13]. This study was conducted with the assumption that the eradication of H.pylori can be helpful in children with positive CD serology and reduce the amount of common serological tests despite the continuous consumption of gluten.

Therefore, the purpose of this study is to investigate the effect of eradication of H.pylori infection on the serum level of common serological tests, including IgA anti tTG.

## Materials and methods

### Data source and subjects

This study was designed to explain the probable involvement of H. pylori in mimicking celiac disease pathology and, as a result, influencing screening test results. To do so, we selected three groups of children between 2 and 18 years old were referred to Mofid children hospital and Children’s Medical Center, tertiary medical centers of children in Tehran/Iran for diagnosis of CD.

IgA EMA was measured by an indirect immunofluorescence method ((EmA Kit Biosystems, Genova, Italy). IgA anti TTG was measured by ELISA (ImmuLisa, Immco, USA).

Patients referred for upper endoscopy for confirming diagnosis of CD. Five biopsy samples (4 from D2 and 1 from bulb) were sent. In addition four gastric biopsy samples were sent (2 from antrum, 1 from cardia and 1 from body). Two pathologists expert in the field have reviewed the tissues.

The two groups who had moderate to heavy colonization with H.pylori infection according to gastric biopsy results. These patients introduced to ethics committee of research institute for children health. After written informed consent they referred to pediatric gastroenterologist for treatment of H pylori infection. Two weeks course of treatment with antibiotics and proton pomp inhibitor was performed. All patients had examined for H.pylori infection eradication by stool antigen 6 weeks later. During these 6 weeks, the patients were not subjected to a gluten-free diet, but after the 6th week and re-examination and eradication of H.pylori infection in the stool, based on the confirmation of celiac disease, they were subjected to the desired diet. Furthermore, the anti-endomysial antibody (EMA-IgA(mg/dL)) test was checked before and after therapy in the first group. This test was also performed in the third group of celiac patients with negative H. pylori, but not in the second group of non-celiac patients with positive H.pylori because it was not scientifically necessary.

### Statistical analysis

The normality of the data was initially assessed in order to select the suitable test. If the data between the two groups were normal, a paired or unpaired T-Test was used; if they were not normal, Mann-whitney or Wilcoxon tests were used. For comparisons between more than two groups, either analysis of variance or the Kruskal-Wallis test was considered. In all cases, the significance level was less than 0.05.

## Results

**Fig. 1:**
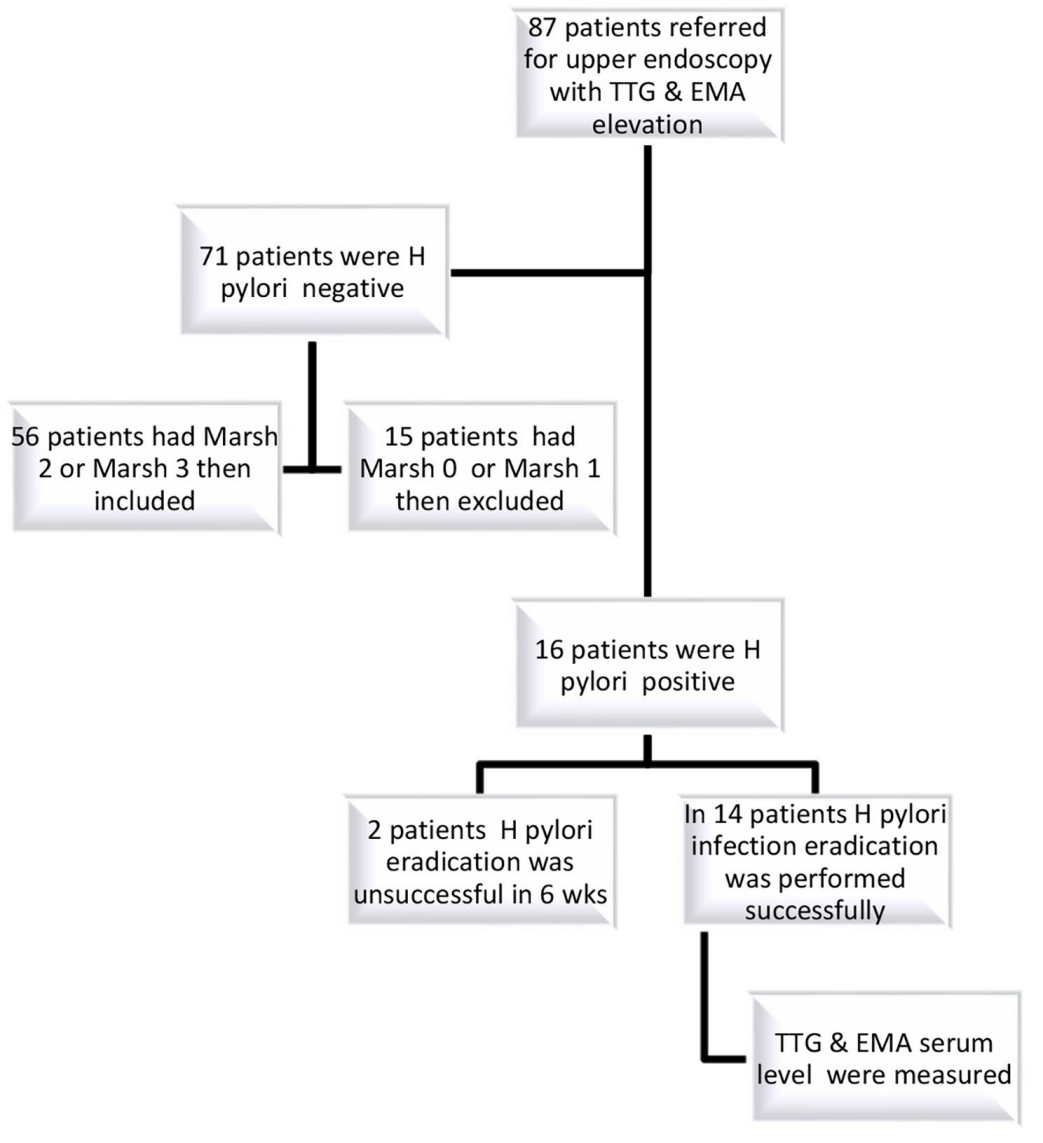
Study summery

This study was conducted between 2021 and 2022 and on children aged 2–18 years who referred to children’s hospital. The first group included 16 CD patients who tested positive for H. pylori. The second group included 16 children who had H. pylori but did not have CD. The third group included 56 celiac disease patients who tested negative for H.pylori. In the first group of our samples (positive H. pylori, celiac patients), the mean age of the subjects was 9.7 ± 3.33 years. In the second group (positive H. pylori, non-celiac patients), the mean age of the subjects was 11.8 ± 3.14 years, and finally, in the third group (celiac patients, negative H. pylori) the mean age was 7.6 ± 3.32 years. In the first group, nine children were girls and seven were boys. In second group, nine children were boys and seven were girls. In third group, 26 children were boys and 30 were girls (Fig. 1). As shown in Table 1, the histological distributions of the subjects in groups one and three were almost similar so they often had marsh 2 or/and 3. The histological distribution of two groups in the March 3 class was also no different (chi2 = 6.0, p = 0.199).

**Table 1:** Histology findings of patients in group 1 and 3

*Group*	*Marsh 1*	*Marsh 2*	*Marsh 3*
***One (n = 16)***	*0*	*5*	* A = 6, B = 5, C = 0*
***Three (n = 56)***	*0*	*6*	* A = 18, B = 24, C = 8*

### Celiac patients with positive H. pylori (group one)

**Fig. 2:**
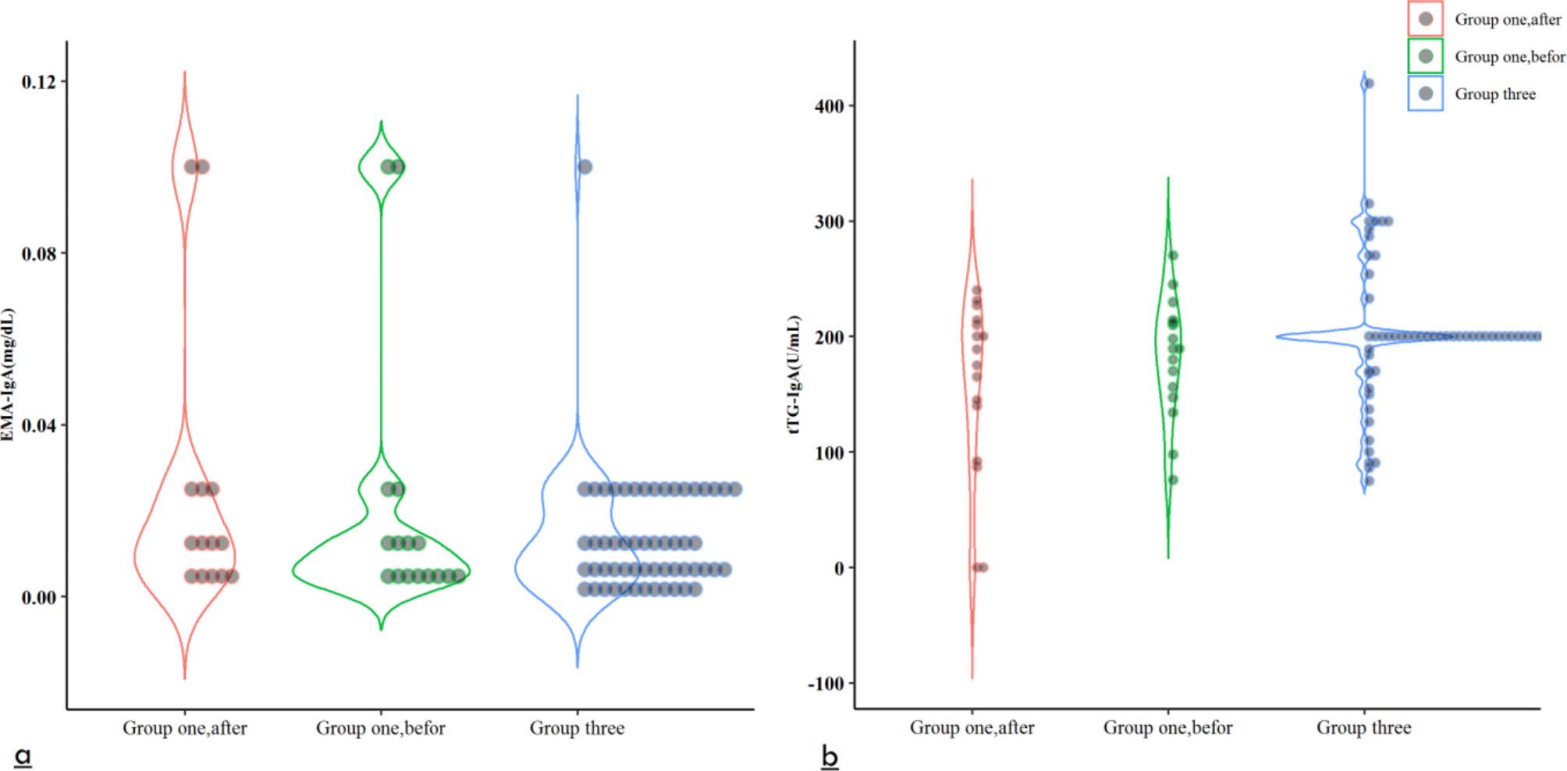
Mean tTG-IgA and EMA-IgA levels in the groups one and three before and after Helicobacter pylori eradication

The mean tTG value after eradication of Helicobacter pylori was higher than before eradication (182.43 vs. 157.18, P = 0.121) which was not statistically significant (Table 2; Fig. 2b). Conversely, EMA mean score was lower after eradication (0.021 vs. 0.022, P = 0.831) and was not significant (Table 2; Fig. 2a). The mean tTG in boys and girls after treatment was lower than before treatment (girls, 150.7 vs. 170.2 and boys, 165.4 vs. 198.1) which was not significant. Subsequently, the mean EMA was higher in girls after eradication but lower in boys (girls, 0.023 vs. 0.021 and boys, 0.0196 vs. 0.0205). These findings might point to a possible involvement for H. pylori in the findings of two screening tests, tTG and EMA, and, ultimately, the diagnosis of celiac disease. However, it can also show a gender-confusing role.

**Table 2:** Mean tTG-IgA and EMA-IgA levels among study groups before and after Helicobacter pylori eradication

	*tTG-IgA(U/mL)*		*P-value*	*EMA-IgA(mg/dL)*		*P-value*
*Group*	*Before*	*After*		*Before*	*After*	
***One***	*157.18(76.28)*	*182.43(51.46)*	*0.121*	*0.022(0.031)*	*0.021(0.031)*	*0.831*
***Two***	*22.18(56.21)*	*9.56(8.27)*	*0.449*	*-*	*-*	*-*
***Three***	*202.50(63.06)*	*-*	*-*	*0.014(0.014)*	*-*	*-*

Data are reported as mean (SD).

### Non-Celiac patients with positive H. pylori (group two)

**Fig. 3:**
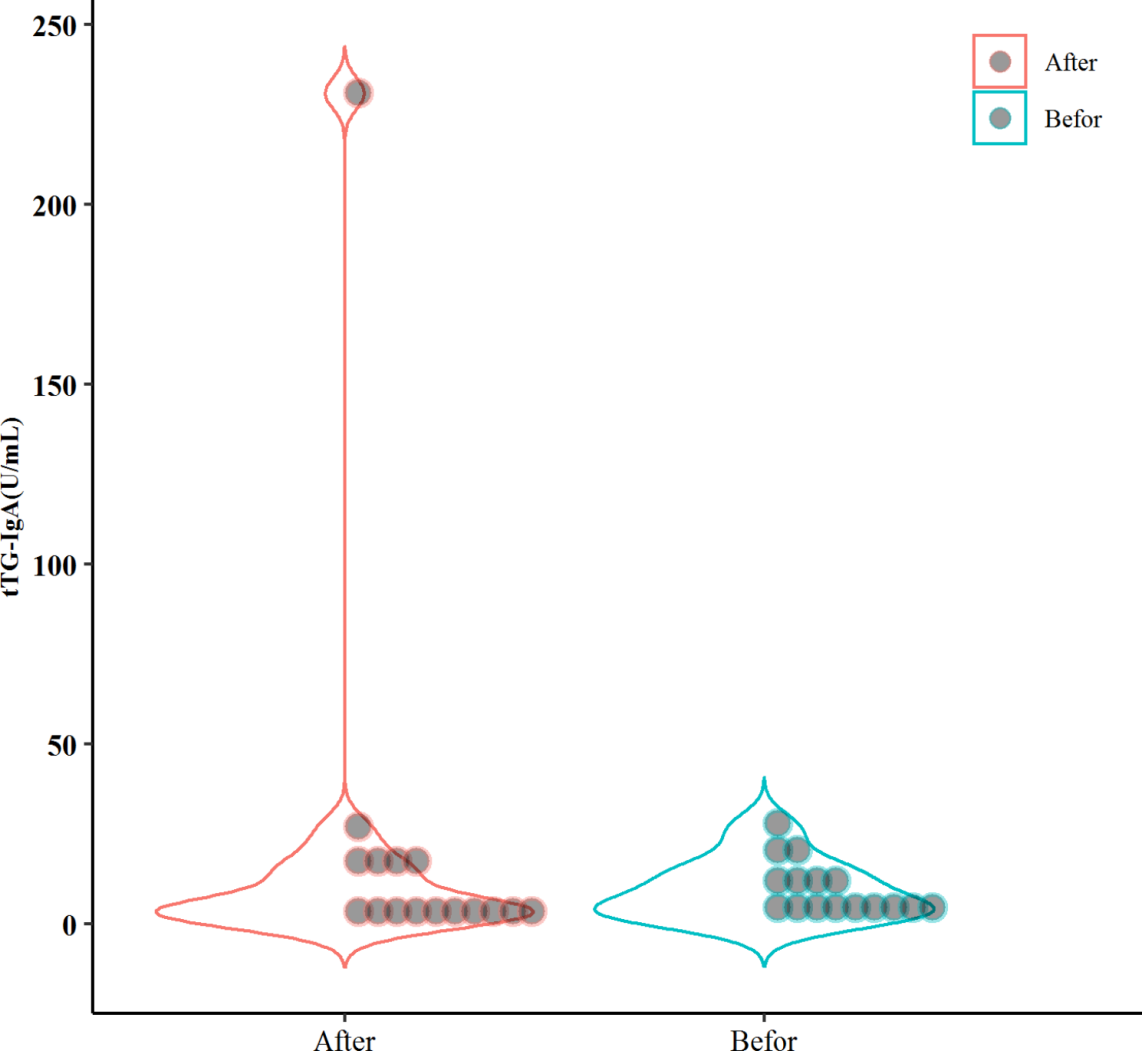
Mean tTG-IgA levels in the group two before and after Helicobacter pylori eradication

In this case, the mean amount of tTG after H. pylori eradication was lower than before eradication (9.56 vs. 22.18, P = 0.449), although the difference was not statistically significant as demonstrated in Table 2; Fig. 3. The mean level of tTG after treatment for H. pylori eradication was lower in girls (11.28 vs. 12) but greater in boys (30.66 vs. 7.66). Similarly, these findings might point to an involvement for H. pylori as well as a sex-confounding role.

### Celiac patients with negative H. pylori (group three)

At the baseline level, the mean tTG in this group was closer to the mean tTG in the first group (202.50 vs. 182.43) Table 2; Fig. 2a. This similarity might reassure us about H. pylori’s probable involvement in this test. This group’s mean EMA value was 0.014, which differed from the first group’s values both before and after treatment Table 2; Fig. 2b.

## Discussion

In recent years, significant progress has been made in improving the diagnosis of CD with the development of new diagnostic tools. Although it is still the use of celiac-related antibody detection tests as the first step of screening in symptomatic patients [3], however, the increase in serum levels of anti-tTG in various diseases and conditions such as autoimmune diseases, diabetes mellitus, infections, tumors, injuries myocardium, liver disorders, and psoriasis are observed without any evidence of CD, which can be an obstacle for the differential diagnosis of CD and make its diagnosis challenging [14–19]. On the other hand, it has been shown that the histopathological changes of CD may be associated with various conditions, including intestinal infections, drugs, autoimmune enteropathy, immunodeficiency, eosinophilic enteropathy, and small intestinal bacterial overgrowth, and these changes are nonspecific. Some findings also indicate an increase in intra-epithelial lymphocytes in the duodenum with a normal villi structure, which is often associated with H. pylori gastritis and may improve with the eradication of this infection [20–23]. In previous studies, a cause-and-effect relationship between this infection and CD has been reported, however, this evidence is limited to epidemiological studies and has resulted in contradictory results, which can be caused by the difference in the type of diagnostic method and the lack of proper selection of the control group [6, 12, 24–26].

Our results showed that in group 1 (patients with celiac disease and positive for H.pylori), mean tTG increased after eradication of H.pylori infection, however, these changes were not significant. In the second group (non-Celiac patients with positive H. pylori), although unlike the first group, mean tTG decreased after eradication of the infection, but still these changes were not significant. The observation of an increase in mean tTG in the first group after eradication can support the hypothesis that H.pylori infection has a protective role against CD, so that this hypothesis has been mentioned in several studies [27–29] and the results observed in the third group of our study can also show this issue. In group 3, it showed that at the baseline level, the mean tTG in this group was closer to the mean tTG in the first group (202.50 vs. 182.43). However, these results and changes were not significantly reported in our study, and in other studies, this hypothesis was contradictory and cannot confirm this result. However, the results reported from the second group contradicted this hypothesis and the results of the second group and indicated the small effect of eradicating this infection in reducing mean tTG. A recent study has also been conducted in this regard [30]. So that in line with our results in non-celiac patients who tested positive for H.pylori, in 80% of these patients the mean tTG level significantly decreased after treatment of this infection with follow-up 6 month [30]. However, the observation of non-significant results in our study can be due to the small subjects and the small follow-up of patients, and on the other hand, different tests and diagnostic kits have been used to test positive for H.pylori infection and tTG which can affect the results. Also, the difference in the follow-up of patients can be another reason for explaining this contradiction in the results. Furthermore, In the study of Akkelle et al. [30], unlike our study, all patients with CD were subjected to a gluten-free diet, which can significantly affect the results and cause a decrease in mean tTG in people with CD and with a positive H. pylori test after treatment of this infection. In another study conducted in 2020 by Gungor et al [31]. in order to link between H.pylori and CD in pediatrics, contradicting our results, the findings showed that after the eradication of H.pylori, okutransglutaminas antibody level (DTG) and EMA) serology significantly decreased in children with CD potential. However, in the mentioned study, DTG test was used instead of tTG, which seems to have a different sensitivity than glucose. On the other hand, in Gungor’s study, significant findings were observed after eradication of H.pylori in children with celiac potential that according to the author’s statement, tissue biopsy has not yet confirmed celiac disease, which can affect the findings.

Our findings also indicated a gender-dependent role in mean tTG levels. So that in some studies, it confirms these results [32]. So that in one study, it was observed that female patients with CD have significantly higher autoantibody titers compared to male patients [32] and in various studies, the role of sexual bias in the sensitivity and severity of autoimmune diseases is well known [33]. In addition, the prevalence of CD disease is more common in women and its clinical manifestations are more severe and faster [34]. In animal models, it shows that this gender difference in autoimmune diseases can be caused by a TH1 or TH2 helper lymphocyte, which ultimately causes a stronger immune response and thus more antibody production in women [33].

Among the strengths of this study was the use of valid diagnostic tests to diagnose H.pylori infection and CD, including histological, and endoscopic evidence. On the other hand, we tried to consider all necessary potential include and exclude criteria and all patients entered the study with full knowledge and consent. In addition, the data related to this study was collected from a reference hospital to increase the generalizability of the results and to minimize the confounding factors regarding the patients. However, the small sample size and the short duration of the intervention were among the limitations of our study. One of the reasons for the short duration of the intervention was to comply with the ethics of patients with CD who were included in the study without common treatment during the study. Although all patients with CD entered the study with full consent and according to common guidelines, 6 weeks of not receiving common treatment has no effect on any of the disease factors [3]. Another limitation of this study was the lack of adjustment for potential confounders such as individual characteristics of patients and genetic factors. Furthermore, IgA anti-tTG test is a highly sensitive screening test for celiac disease that it specifically detects the gliadin peptides in the blood. In case there is an H. pylori infection, and it entangles with gluten peptides, we believe the IgA anti-tTG test will not confuse it. However, in such conditions, a novel microbial tTG (mTG) test can be performed that is specially designed for this purpose.

## Conclusion

Our findings generally showed that the eradication of H.pylori infection does not have a significant effect on tTG levels in any of the studied groups in children with and without CD. However, more studies and better design and longer intervention duration are needed to investigate these findings.

## Data Availability

Data is available upon request from the corresponding author for the article due to privacy / ethical restrictions.
